# The Co-Expression Pattern of Calcium-Binding Proteins with γ-Aminobutyric Acid and Glutamate Transporters in the Amygdala of the Guinea Pig: Evidence for Glutamatergic Subpopulations

**DOI:** 10.3390/ijms241915025

**Published:** 2023-10-09

**Authors:** Daniel Kalinowski, Krystyna Bogus-Nowakowska, Anna Kozłowska, Maciej Równiak

**Affiliations:** 1 Department of Animal Anatomy and Physiology, Faculty of Biology and Biotechnology, University of Warmia and Mazury in Olsztyn, pl. Łódzki 3, 10-727 Olsztyn, Poland; boguska@uwm.edu.pl (K.B.-N.); mrowniak@uwm.edu.pl (M.R.); 2 Department of Human Physiology and Pathophysiology, School of Medicine, University of Warmia and Mazury in Olsztyn, Warszawska 30, 10-082 Olsztyn, Poland; kozlowska.anna@uwm.edu.pl

**Keywords:** amygdala, calcium-binding proteins, VGAT, VGLUT2, immunohistochemistry, guinea pig

## Abstract

The amygdala has large populations of neurons utilizing specific calcium-binding proteins such as parvalbumin (PV), calbindin (CB), or calretinin (CR). They are considered specialized subsets of γ-aminobutyric acid (GABA) interneurons; however, many of these cells are devoid of GABA or glutamate decarboxylase. The neurotransmitters used by GABA-immunonegative cells are still unknown, but it is suggested that a part may use glutamate. Thus, this study investigates in the amygdala of the guinea pig relationships between PV, CB, or CR-containing cells and GABA transporter (VGAT) or glutamate transporter type 2 (VGLUT2), markers of GABAergic and glutamatergic neurons, respectively. The results show that although most neurons using PV, CB, and CR co-expressed VGAT, each of these populations also had a fraction of VGLUT2 co-expressing cells. For almost all neurons using PV (~90%) co-expressed VGAT, while ~1.5% of them had VGLUT2. The proportion of neurons using CB and VGAT was smaller than that for PV (~80%), while the percentage of cells with VGLUT2 was larger (~4.5%). Finally, only half of the neurons using CR (~53%) co-expressed VGAT, while ~3.5% of them had VGLUT2. In conclusion, the populations of neurons co-expressing PV, CB, and CR are in the amygdala, primarily GABAergic. However, at least a fraction of neurons in each of them co-express VGLUT2, suggesting that these cells may use glutamate. Moreover, the number of PV-, CB-, and CR-containing neurons that may use glutamate is probably larger as they can utilize VGLUT1 or VGLUT3, which are also present in the amygdala.

## 1. Introduction

The amygdala is an almond-shaped set of brain nuclei located deep in the temporal lobe, which is critically important for the regulation of various aspects of emotion, including fear, anxiety and aggression processing, emotional learning and memory, and social cognition [1,2,3]. In addition, it actively participates in appetite conditioning, sexual behavior, drug addiction, and stress response [4,5,6,7].

In general, the amygdala is a highly diversified brain structure composed of ~13 different cell groups, which together form quite distinct two larger regions [8,9,10]. These two regions are differentiated parts of the telencephalon with different embryological origins [8,9,11] and cellular structure [3,12]. The first of these regions is the “cortex-like” amygdala due to pallial origin [8,9,11]. This region includes the basolateral complex (BLC) comprising the lateral (LA), basolateral (BL), and basomedial (BM) nuclei, as well as the cortical nuclei (CO) and the nucleus of the lateral olfactory tract (NLOT) [8,9]. Although there is a general consensus in the literature that all these nuclei are pallial derivatives, the exact origin of this region is still under debate [8,13,14]. They may be the embryonic claustrum, the lateral pallium, or the ventral pallium [8,13,14]. Despite the fact of this unresolved embryological origin, the “cortex-like” nuclei are organized in a very similar way. They have a similar cellular structure, with two major cell classes, such as pyramidal or pyramidal-like neurons and non-pyramidal neurons [3,12]. The pyramidal or pyramidal-like neurons, as in the cortex, are projection neurons, which use glutamate as an excitatory neurotransmitter [15] and constitute ~80% of the whole cell population [16,17]. Most non-pyramidal neurons, similar to the cortex, are interneurons [18], which use γ-aminobutyric acid (GABA) as an inhibitory neurotransmitter [19,20] and constitute ~20% of the neuronal population [19]. The second of these regions is the “striatum-pallidum-like” amygdala due to subpallial origin [8,13,14]. This region is represented primarily by the central nucleus (CE) and the medial nucleus (ME), but it also includes the anterior amygdaloid area (AAA) and intercalated cell masses [8,11,13]. The “striatum-pallidum-like” amygdala is organized in quite a different way from that of the “cortex-like” amygdala. It is populated primarily by GABAergic neurons [8,10,21]. However, glutamatergic neurons, although less numerous, also are present there [22]. Moreover, while in the “cortex-like” amygdala, most projection neurons use glutamate as a fast, excitatory neurotransmitter [15], whereas, in contrast, many projection cells of the “striatum-pallidum-like” amygdala use GABA as a fast, inhibitory neurotransmitter [8,23,24]. In the “cortex-like” amygdala, glutamate decarboxylase (GAD) is usually expressed in interneurons [25,26]. Whereas in the “striatum-pallidum-like” amygdala, it is often expressed in projection neurons [8,23,24].

Both “cortex-like” and “striatum-pallidum-like” amygdala have large populations of neurons containing calcium-binding proteins (CaBPs), such as parvalbumin (PV), calbindin (CB), or calretinin (CR) [19,27]. Although there is general consent that in the “cortex-like” amygdala, PV, CB, and CR-containing cells are primarily GABAergic interneurons [28]. However, it seems that not all these cells use GABA [19,29], and not all of them are interneurons [30,31]. At least a part of neurons containing PV, CB, or CR in the basolateral amygdala are GABA-immunonegative [17,19]. Moreover, there is also evidence that some CB- and CR-expressing neurons in the basolateral amygdala project to the extrinsic brain regions [30,31]. In the “striatum-pallidum-like” nuclei such as CE and ME, data on the co-expression nature of neurons containing calcium-binding is lacking. These nuclei are devoid of PV-expressing neurons, but they are rich in CB- or CR-containing cells [19,27]. They have many GABA-projecting neurons [8,23,24]. However, glutamatergic neurons are also present there [22]. Thus, at least a part of CB- or CR-containing cells in the “striatum-pallidum-like” amygdala may be GABAergic projection neurons, but some of these cells may also use glutamate. Finally, there is one more intriguing and still unresolved issue. There is no evidence that any of the cells containing PV, CB, or CR in the amygdala may contain glutamate yet. However, the neurons containing PV or CR and glutamate have been demonstrated in the cerebral cortex [32,33], which has anatomical and electrophysiological features like that in the “cortex-like” amygdala [34,35,36]. Moreover, in the basal forebrain, while most PV neurons are GABAergic, a larger portion of CB or CR cells are glutamatergic, not GABAergic [37].

Taken together, in the amygdala are two large regions with different embryological origins and cellular structures: “cortex-like” and “striatum-pallidum-like” amygdala. Both these regions have populations of neurons using GABA and glutamate, and each of them is rich in cells containing PV, CB, or CR. Although relationships between GABA and PV, CB, or CR in the “cortex-like” amygdala are quite well documented [19], these relationships in the “striatum-pallidum-like” regions are still obscure. Moreover, such relationships between glutamate and PV, CB, or CR in each of the regions in the amygdala have never been investigated. Considering that “cortex-like” and “striatum-pallidum-like” amygdala have different origins, the populations of PV-, CB- or CR-expressing neurons also may have quite different origins, and their co-expression patterns with GABA and glutamate may also be different. Thus, the present study investigates for the first time in the amygdala of the guinea pig relationships between the cells containing PV, CB, or CR and either the vesicular GABA transporter (VGAT, marker of GABAergic neurons) [38] or vesicular glutamate transporter type 2 (VGLUT2, marker of glutamatergic neurons) [39]. Even though VGAT and VGLUT2 are present in synaptic vesicles, they are also known to be markers for glutamatergic and GABAergic neurons [40,41,42,43,44,45]. The first definitive marker of glutamatergic neurons was vesicular glutamate transporters [41,42]. Furthermore, the specificity of VGLUT2 in the detection of glutamatergic somata has been established via previous research employing qualified methods [41,45,46,47,48,49,50].

## 2. Results

### 2.1. Distribution Pattern of PV, CB, and CR Neurons in the Amygdala of the Guinea Pig

**Figure 1:**
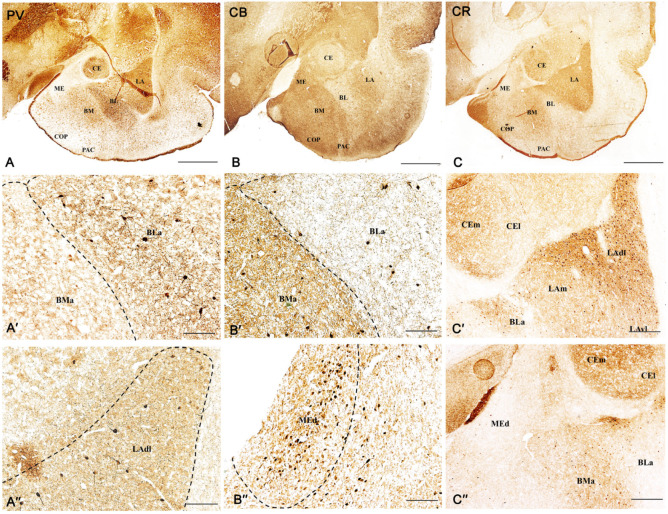
(Modified) All figures were published in Równiak et al. [27]. Brightfield photomicrographs highlighting the patterns of parvalbumin (PV, **A**–**A″**), calbindin (CB, **B**–**B″**), and calretinin (CR, **C**–**C″**) immunoreactivity in the amygdala of the male guinea pig. The scale bar applies to all microphotographs, and it corresponds to the length of 2000 μm (**A**–**C**) and 200 μm (**A′**–**C′**, **A″**–**C″**). LAdl–lateral nucleus, lateral part, LAm–lateral nucleus, medial part, LAvl–lateral nucleus, ventral part, BLa–basomedial nucleus, anterior part, BMa–basomedial nucleus, anterior part, MEd–medial nucleus, dorsal part, CEm–central nucleus, medial part, CEl–central nucleus, lateral part, COP–cortical nucleus, PAC–piriform-amygdalar area. Noted that nuclei are indicated by the dotted line.

The position of the various nuclei in the amygdala of the guinea pig, as well as the distribution pattern of PV, CB, and CR neurons in this region observed in the present study, closely reflect those described in the guinea pig in our previous reports (Figure 1) [27,51]. Furthermore, the distribution pattern of PV, CB, and CR neurons observed in the guinea pig is quite like that described previously in the rat [17,19], monkey [52], and human [53,54,55]. In the present study, the same six main amygdala regions, i.e., the lateral, basolateral, basomedial, cortical, central, and medial, were chosen for detailed investigation. In all these regions, relationships between PV, CB, or CR neurons and VGAT or VGLUT2 were investigated (Figures in below).

### 2.2. Distribution Pattern of VGAT and VGLUT2 Immunoreactivity in the Amygdala of the Guinea Pig

**Figure 2:**
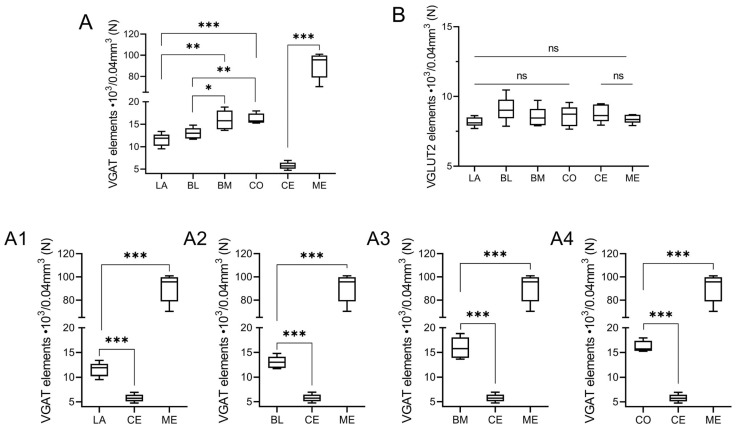
The volume density of vesicular GABA transporter (VGAT, **A**) and vesicular glutamate transporter type 2 (VGLUT2, **B**) in the “cortex-like” and “striatum-pallidum-like” amygdala of the guinea pig (n = 5). Note significant differences between “cortex-like” and “stratum-pallidum-like” nuclei (**A1**–**A4**). Data are expressed as box-and-whiskers plots, with the “box” depicting the median and the 25th and 75th quartiles and the “whiskers” showing the 5th and 95th percentile. * (*p* ≤ 0.05), ** (*p* ≤ 0.01), and *** (*p* ≤ 0.001) indicate statistically significant differences between studied nuclei analyzed by Student’s t-test, whereas ns means none statistically significant. Elements–immunoreactive somata, fibers, and neuropil. LA—lateral nucleus, BL—basolateral nucleus, BM—basomedial nucleus, CO—cortical nucleus, CE—central nucleus, ME—medial nucleus.

VGAT and VGLUT2 immunoreactivity was extensively represented in the whole guinea pig amygdala, as confirmed by automated line scan (Figure 2), and in all the nuclei studied consisted of immunoreactive elements represented by puncta, fibers, and somata (Figures in below). 

**Figure 3:**
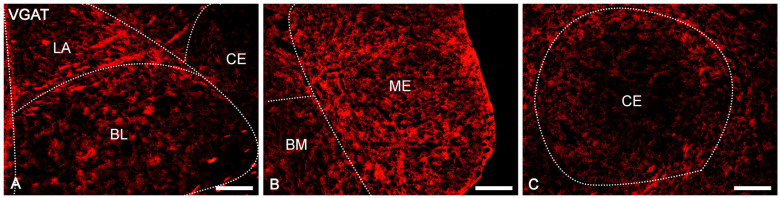
Representative color photomicrographs illustrating immunoreactivity of vesicular GABA transporter (VGAT, **A**–**C**) in the amygdala of guinea pig. The scale bar applies to all microphotographs, and it corresponds to the length of 200 μm. LA—lateral nucleus, BL—basolateral nucleus, BM—basomedial nucleus as a “cortex-like” amygdala, CE—central nucleus, ME—medial nucleus as a “striatum-pallidum-like” amygdala. Noted that nuclei are indicated by the dotted line.

**Figure 4:**
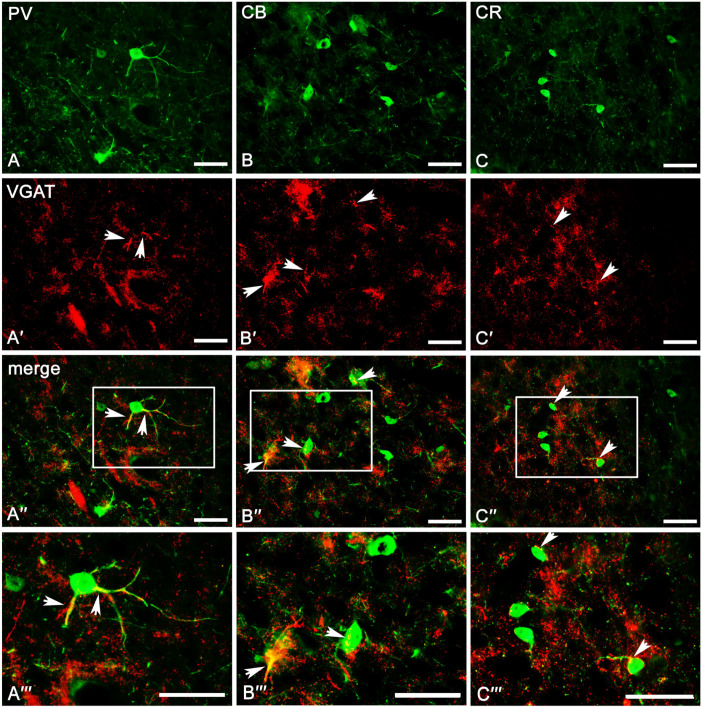
Representative color photomicrographs illustrating the co-expression pattern of PV (**A**–**A‴**), CB (**B**–**B‴**), and CR (**C**–**C‴**) neurons with VGAT in the basolateral (**A**–**A‴**), basomedial (**B**–**B‴**) and cortical (**C**–**C‴**) nuclei as a “cortex-like” amygdala of the guinea pig. Arrows indicate double-labeled cells. Boxed regions in (**A″**–**C″**) are shown at a greater magnification in (**A‴**–**C‴**). The scale bar applies to all microphotographs, and it corresponds to the length of 50 μm.

**Figure 5:**
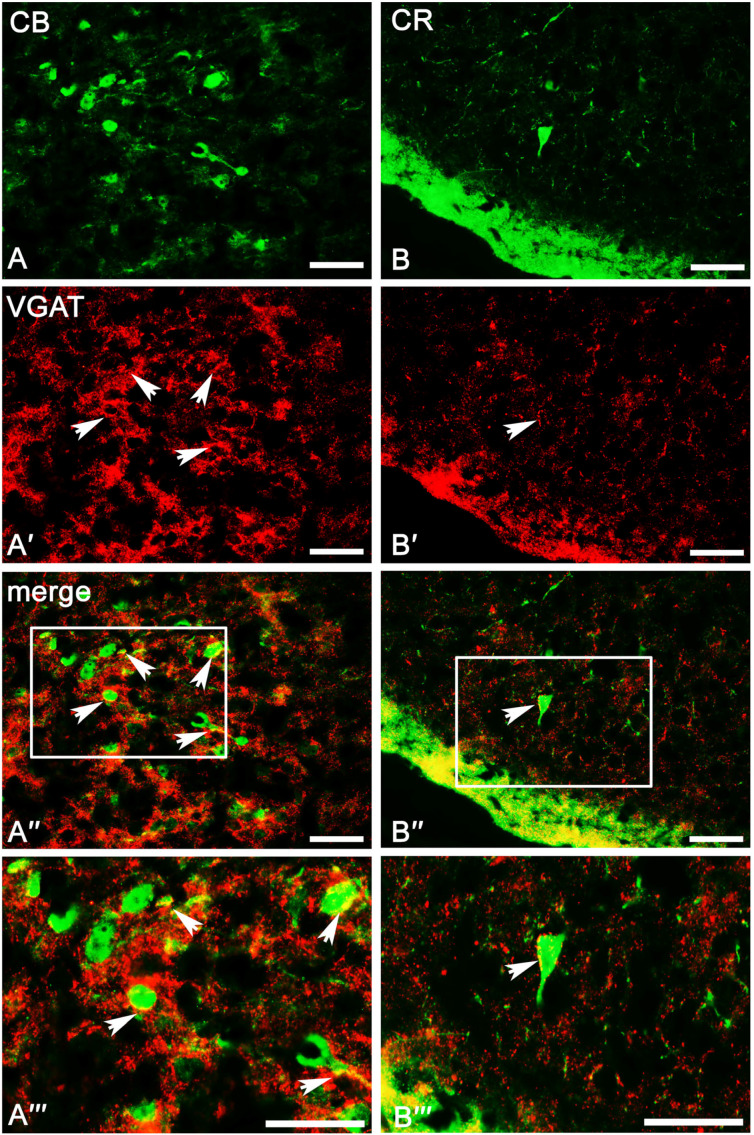
Representative color photomicrographs illustrating the co-expression pattern of CB (**A**–**A‴**) and CR (**B**–**B‴**) neurons with VGAT in the medial nucleus (**A**–**B‴**) as a “striatum-pallidum-like” amygdala of the guinea pig. Arrows indicate double-labeled cells. Boxed regions in (**A″**,**B″**) are shown at a greater magnification in (**A‴**,**B‴**). The scale bar applies to all microphotographs, and it corresponds to the length of 50 μm.

The overall level of VGAT immunoreactivity was very high in the entire amygdala. However, in the “striatum-pallidum-like” amygdala, it was higher, especially in the ME, than in the “cortex-like” amygdala (t_1.28_ = −3.40, *p* = 0.002, Figure 2A(A1–A4) and Figure 3A–C). In addition, within the “cortex-like” amygdala, the level of VGAT immunoreactivity was lower in the LA and the dorsal part of BL when compared to that in the ventral part of BL, BM, and CO (t_1.23_ = −3.61, *p* = 0.0015, Figure 2A and Figure 3A–C). The “striatum-pallidum-like” amygdala was characterized primarily by numerous and intensely stained immunoreactive puncta, densely arranged and extensively labeled VGAT fibers, and the presence of intensely labeled VGAT signal outlining neurons (Figure 3A–C, Figure 4A′–C′ and Figure 5A′–B′). In addition, in the ME (Figure 2A and Figure 3B), fibers were much more numerous and densely packed than in the CE (Figure 2A and Figure 3C). In contrast, in the “cortex-like” region, immunoreactive elements (puncta and fibers) of VGAT were packed looser and characterized by lower intensity of staining than those in the “striatum-pallidum-like” region, especially in the ME (Figure 2A and Figure 3A–C). Furthermore, the VGAT signal around perikaryon was less intense in the “cortex-like” than in the “striatum-pallidum-like” amygdala, which is strictly correlated with its immunoreactivity (Figure 2A(A1–A4) and Figure 3A–C).

**Figure 6:**
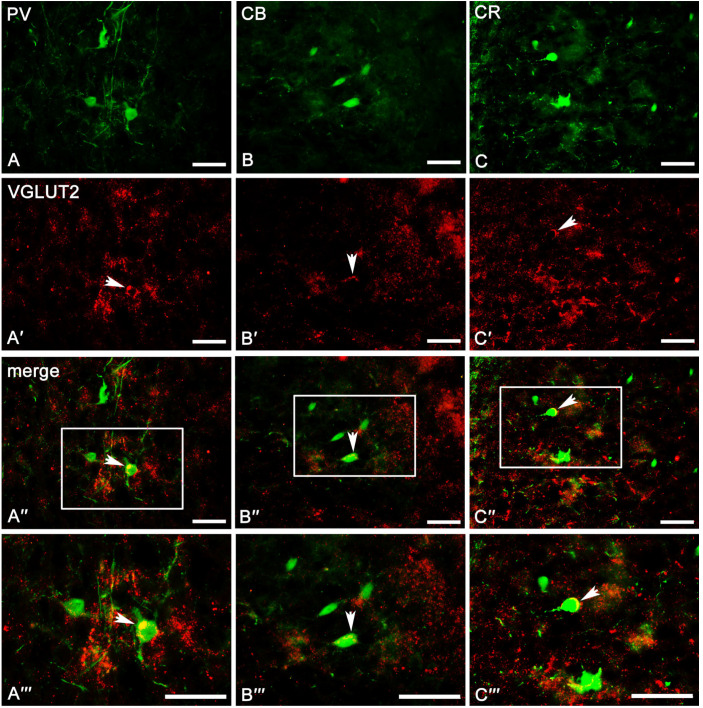
Representative color photomicrographs illustrating the co-expression pattern of PV (**A**–**A‴**), CB (**B**–**B‴**), and CR (**C**–**C‴**) neurons with VGLUT2 in the basolateral (**A**–**A‴**,**C**–**C‴**) and cortical (**B**–**B‴**) nuclei as a “cortex-like” amygdala of the guinea pig. Arrows indicate double-labeled cells. Boxed regions in (**A″**–**C″**) are shown at a greater magnification in (**A‴**–**C‴**). The scale bar applies to all microphotographs, and it corresponds to the length of 50 μm.

**Figure 7:**
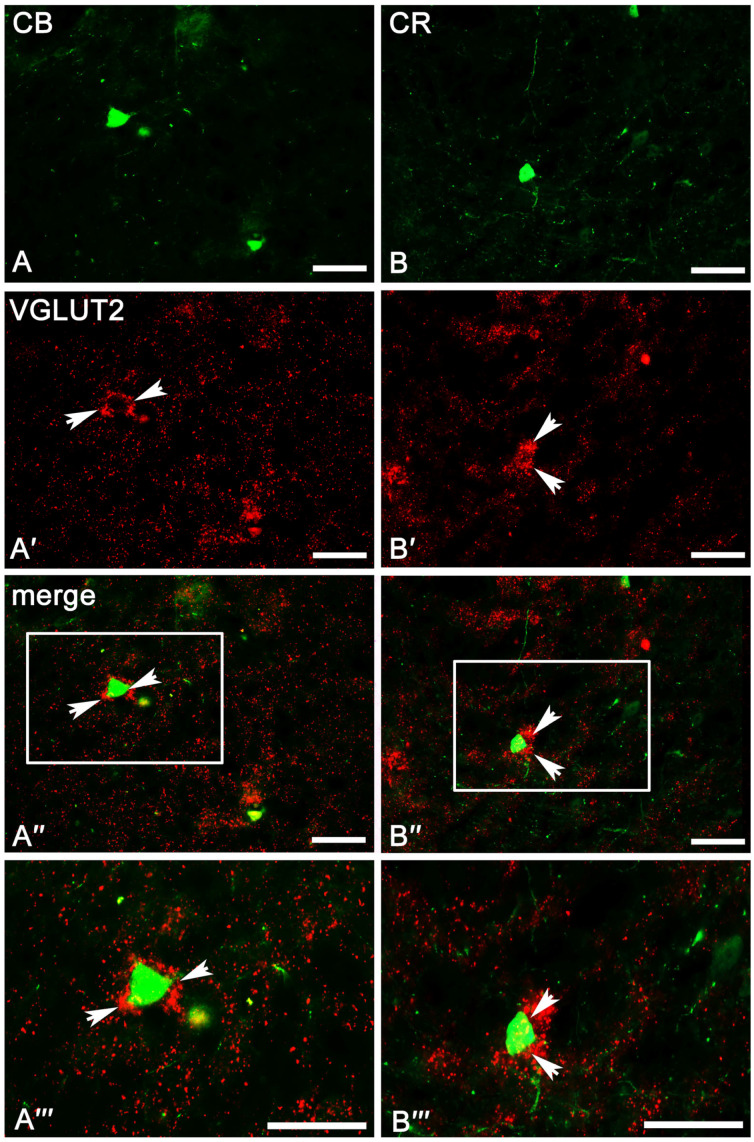
Representative color photomicrographs illustrating the co-expression pattern of CB (**A**–**A‴**) and CR (**B**–**B‴**) neurons with VGLUT2 in the central (**A**–**A‴**) and medial (**B**–**B‴**) nuclei as a “striatum-pallidum-like” amygdala of the guinea pig. Arrows indicate double-labeled cells. Boxed regions in (**A″**,**B″**) are shown at a greater magnification in (**A‴**,**B‴**). The scale bar applies to all microphotographs, and it corresponds to the length of 50 μm.

In the case of VGLUT2, the overall level of immunoreactivity in the whole amygdala was lower than that for VGAT. Furthermore, the intensity of staining and distribution of immunoreactive elements was rather homogenous in the entire amygdala, without evident differences between various amygdala nuclei and between “cortex-like” and “striatum-pallidum-like” regions (Figure 2B). In all studied nuclei, VGLUT2 immunoreactivity consisted mostly of numerous immunoreactive puncta (Figure 6A′–C′ and Figure 7A′–B′). However, signal outlining neurons and fibers, although much less numerous than in the case of VGAT, were present.

### 2.3. Co-Expression Pattern of PV, CB, and CR Neurons with VGAT and VGLUT2 in the Amygdala of the Guinea Pig

The result clearly demonstrates that PV (Table 1), CB (Table 2), and CR (Table 3) neurons have different and unique patterns of co-expression with VGAT and VGLUT2 (Figure 2A–B and Figure 4A″–C‴, Figure 5A″–C‴, Figure 6A″–C‴, Figure 7A″–C‴). The vast majority of PV, CB, and CR neurons primarily co-express VGAT, albeit each of these populations has a smaller or larger fraction of cells co-expressing VGLUT2. These populations arranged according to the largest number of cells co-expressing VGAT are PV (Table 1), CB (Table 2), and finally CR (Table 3). Nevertheless, the largest number of cells co-expressing VGLUT2 are CB, then CR, and finally PV.

#### 2.3.1. Parvalbumin Neurons

Almost all PV neurons in the amygdala co-expressed VGAT (87.4 ± 1.6%, Table 1), and this population had the smallest fraction of cells co-expressing VGLUT2 (1.7 ± 0.2%, Table 1). Moreover, in most cases, the immunoreactivity of VGAT was strong enough to indicate that PV neurons contain high levels of these proteins within the lateral, basolateral, basomedial, and cortical nuclei. Almost 90% of PV neurons co-expressed VGAT (Table 1, Figure 4A–A‴), while VGLUT2 (Figure 6A–A‴) was observed usually in less than 2% of these cells (Table 1). Within the central and medial nuclei, there were no neurons double-labeled for PV and VGAT and/or VGLUT2 due to the limited number of PV cells in both these regions.

**Table 1:** The co-expression pattern of parvalbumin (PV) neurons with VGAT and VGLUT2 in the amygdala of the guinea pig. Note extensive co-localization of parvalbumin-positive neurons with VGAT and very rare associations of these cells with VGLUT2. Data were expressed as mean ± standard error of mean (SEM).

Parvalbumin																		
	VGAT									VGLUT2								
	PV			PV/VGAT			%			PV			PV/VGLUT2			%		
LA	120.3	±	6.7	106.3	±	4.2	88.0	±	1.9	128.0	±	3.5	1.0	±	0.2	1.5	±	0.1
BL	130.7	±	9.9	114.7	±	3.3	87.7	±	2.9	127.7	±	6.9	1.3	±	0.2	1.6	±	0.4
BM	110.0	±	4.4	96.0	±	2.9	86.2	±	0.1	124.0	±	2.0	2.0	±	0.2	1.5	±	0.2
CO	151.0	±	13.5	131.3	±	2.9	87.5	±	1.6	142.3	±	5.7	2.7	±	0.2	2.4	±	0.1
CE	-	-	-	-	-	-	-	-	-	-	-	-	-	-	-	-	-	-
ME	-	-	-	-	-	-	-	-	-	-	-	-	-	-	-	-	-	-
Mean	128	±	8.6	112.1	±	3.3	87.4	±	1.6	130.5	±	4.5	1.8	±	0.2	1.7	±	0.2

#### 2.3.2. Calbindin Neurons

A substantial proportion of CB neurons in the amygdala that co-expressed VGAT (78.2 ± 2.0%, Table 2) was similar to PV (87.4 ± 1.6%, Table 1). However, the percentage of co-expression CB/VGAT was smaller than that for PV/VGAT. Simultaneously, the percentage of CB neurons co-expressing VGLUT2 (4.3 ± 0.4%, Table 2) was usually three times larger than that for PV (1.7 ± 0.2%, Table 1). Double-labeled neurons for CB and VGAT (Figure 4B–B‴ and Figure 5A–A‴) and VGLUT2 (Figure 6B–B‴ and Figure 7A–A‴) were observed in all the nuclei studied, albeit the percentages differed among various regions. For example, within the lateral, basolateral, and basomedial nuclei, approximately 75% of CB neurons co-expressed VGAT, but within the cortical nucleus, the percentage of such neurons was even higher, exceeding 80%. In all these regions, VGLUT2 was observed in ~4% of CB cells. Within the central and medial nuclei, double-labeled neurons for CB and VGAT were observed in almost 80% of neurons, while ~6% of CB neurons were co-stained for VGLUT2 (Table 2).

**Table 2:** The co-expression pattern of calbindin (CB) neurons with VGAT and VGLUT2 in the amygdala of the guinea pig. Note that the extent of co-localization of CB neurons with VGAT is smaller than that of PV. Note also that the association of these cells with VGLUT2 is more extensive than that of PV neurons. Data were expressed as mean ± standard error of mean (SEM).

Calbindin																		
	VGAT									VGLUT2								
	CB			CB/VGAT			%			CB+			CB/VGLUT2			%		
LA	117.3	±	3.3	91.7	±	4.1	77.2	±	1.4	121.7	±	0.4	3.0	±	0.6	3.6	±	0.5
BL	160.0	±	2.1	121.0	±	1.0	75.6	±	1.1	192.3	±	3.7	7.3	±	0.4	4.4	±	0.2
BM	133.7	±	5.3	106.3	±	2.4	77.2	±	1.9	146.0	±	4.9	4.0	±	0.4	3.5	±	0.4
CO	176.0	±	4.5	145.0	±	8.0	80.8	±	2.8	159.0	±	7.3	3.3	±	0.8	3.0	±	0.3
CE	131.0	±	8.5	105.0	±	6.5	79.2	±	1.2	142.7	±	3.3	4.3	±	0.4	4.6	±	0.1
ME	263.3	±	21.6	213.0	±	8.0	79.1	±	3.8	230.0	±	1.6	10.7	±	0.4	6.6	±	1.0
Mean	163.6	±	7.6	130.3	±	5.0	78.2	±	2.0	165.3	±	3.5	5.4	±	0.5	4.3	±	0.4

#### 2.3.3. Calretinin Neurons

In contrast to PV (87.4 ± 1.6%, Table 1) and CB (78.2 ± 2.0%, Table 2) neurons, only approximately 50% of CR (52.8 ± 3.5%, Table 3) neurons in the amygdala co-expressed VGAT and the percentage of these cells co-expressing VGLUT2 (3.4 ± 0.1%, Table 3) was usually two times larger than that for PV (1.7 ± 0.2%, Table 1). Within the lateral, basolateral, and medial nuclei, approximately 50% of CR neurons co-expressed VGAT (Figure 4C–C‴ and Figure 5B–B‴), and only within the basomedial and cortical nuclei the percentage of such neurons was higher, exceeding 56%. The percentage of CR neurons co-expressing VGLUT2 (Figure 6C–C‴ and Figure 7B–B‴) in all these regions was in a range from 2% to 6% (Table 3). Within the central nucleus, there were no neurons double-labeled for CR, VGAT, and VGLUT2 due to the limited number of CR cells in this region.

**Table 3:** The co-expression pattern of calretinin (CR) neurons with VGAT and VGLUT2 in the amygdala of the guinea pig. Note that only half of CR-positive neurons co-express VGAT. Note also that the association of these cells with VGLUT2 is more extensive than that of parvalbumin-positive neurons. Data were expressed as mean ± standard error of mean (SEM).

Calretinin																		
	VGAT									VGLUT2								
	CR			CR/VGAT			%			CR			CR/VGLUT2			%		
LA	306.0	±	15.5	152.0	±	6.1	52.6	±	2.9	235.3	±	4.1	6.3	±	0.4	2.4	±	0.0
BL	251.3	±	21.2	118.7	±	1.6	49.2	±	3.7	152.7	±	9.8	5.0	±	0.8	4.2	±	0.2
BM	207.7	±	18.8	119.0	±	18.0	57.0	±	2.7	161.0	±	3.7	2.7	±	0.5	2.3	±	0.1
CO	205.7	±	13.1	114.7	±	3.8	57.6	±	3.7	166.3	±	3.3	6.0	±	0.4	5.5	±	0.2
CE	-	-	-	-	-	-	-	-	-	-	-	-	-	-	-	-	-	-
ME	230.7	±	16.7	102.3	±	16.3	47.6	±	4.4	201.3	±	1.2	2.7	±	0.4	2.4	±	0.1
Mean	240.3	±	17.1	121.3	±	9.2	52.8	±	3.5	183.3	±	4.4	4.5	±	0.5	3.4	±	0.1

## 3. Discussion

To the best of our knowledge, the present study describes for the first time a systematic analysis of relationships between populations of PV, CB, and CR neurons and neurons equipped with vesicular GABA and glutamate transporters (VGAT or VGLUT2). The results show that in the amygdala of the guinea pig, the vast majority of PV, CB, and CR neurons primarily co-express VGAT, albeit each of these populations also has a smaller or larger fraction of cells co-expressing VGLUT2. Thus, the present results confirm previous observations indicating that populations of PV, CB, and CR neurons are primarily GABAergic [17,19]. However, these results also provide the first direct evidence that there is a fraction of neurons in each of these populations that contain VGLUT2, suggesting that these cells may be glutamatergic. It is worth mentioning that the populations of PV, CB, or CR neurons, which may use glutamate in the amygdala, are probably more numerous as in the present study, only VGLUT2 was used as a marker of glutamatergic neurons, and these cells may also utilize VGLUT1 and/or VGLUT3 which are also present there.

The present results demonstrate that in the amygdala of the guinea pig, almost all PV neurons (~90%) co-expressed VGAT, and these neurons had the smallest fraction of cells co-expressing VGLUT2 (~1.5%). Thus, these results are consistent with some previous studies indicating that most PV neurons in the amygdala are GABAergic, which could be an excellent marker for GABAergic neurons [17,19]. For example, Kemppainen and Pitkänen [19] and McDonald and Mascagni [17], using the mirror technique, reported that in the rat basolateral amygdala, ~94% of PV neurons are GABA-positive. Furthermore, Mascagni et al. [16], using double immunofluorescence, evidenced that the extent of co-expression of PV and GABA in the monkey amygdala is quite similar, as it ranged from 90% to 94%. There are many other lines of experimental evidence indicating that PV neurons are primarily inhibitory GABAergic interneurons. The synaptic and electrophysiological studies indicated that most PV neurons are fast-spiking interneurons, which constitute a critical component of the inhibitory circuitry, at least in the “cortex-like” amygdala where they form characteristic basket-like plexuses around the unstained somata and cartridges, representing axon-axonic contacts on initial segments of axons [17,18,56]. Thus, as in the cerebral cortex, many PV interneurons in both the rodent and primate amygdala appear to be basket or chandelier cells that provide a strong perisomatic inhibition of local pyramidal neurons [53,56,57,58]. On the other hand, the present results and all previous co-expression studies [17,19] clearly indicate that although most PV neurons in the mammalian amygdala are GABAergic, at least a small fraction of them do not display GABA markers. The neurotransmitters used by these non-GABAergic PV cells are unknown yet. However, the present study provides evidence that at least part of them co-express VGLUT2, suggesting that they may use glutamate. This suggestion may be partially supported by the fact that neurons containing PV and glutamate have been identified in the cerebral cortex, which is very similar to the various “cortex-like” regions in the amygdala [32]. Moreover, these cortical PV neurons sent their axons to the striatum, indicating that these cells were not interneurons but truly projecting neurons [32]. Similar excitatory cortical PV neurons were also reported in the mouse forebrain [59]. The cells co-expressing PV and VGLUT2 were also observed in the preoptic area [50], which has many embryological relationships with the “striatum-pallidum-like” amygdala.

The present results demonstrate that, similarly to PV neurons, a substantial proportion of CB cells in the amygdala of the guinea pig co-expressed VGAT. However, the percentage of co-expression of both these markers was smaller than that for PV (~80%). Furthermore, the percentage of CB neurons co-expressing VGLUT2 was usually three times larger than that for PV (~4.5%). This is quite congruent with the studies of Kemppainen and Pitkänen [19], who reported that in the rat basolateral amygdala, 75% of CB neurons were GABA-positive. In contrast, [17] reported that 98% of these cells in the rat basolateral amygdala were GABA-positive. Despite this discrepancy, the present results, previous studies [17,19], and synaptic studies indicate that the vast majority of CB neurons are local circuit interneurons that perform a dual role in the intrinsic inhibitory mechanism of the amygdala. In rodents (but not primates), some of them appear to be basket cells, which provide a strong perisomatic inhibition of local pyramidal neurons, and such cells co-express PV but do not contain somatostatin and/or neuropeptide Y [53,56,57,58]. Other CB neurons are dendrite-targeting interneurons, providing a robust innervation of dendrites in the rat and primate amygdala [16]. Such neurons contain somatostatin and/or neuropeptide Y (but not PV) [60,61] and are responsible for the control of the efficacy and plasticity of inputs from specific sources that terminate in the same dendritic domain [62,63]. On the other hand, although the role of CB neurons in the intrinsic inhibitory circuitry of the amygdala is sufficiently proven, there are also some studies evidencing that not all CB cells are interneurons, but at least a part of them are projection neurons. There is direct evidence that some CB neurons in the rat basolateral amygdala project to the extrinsic brain regions [31,64]. Similar cells, which contain CB and project to extrinsic areas, were also identified in the cerebral cortex [32] and hippocampus [65,66]. Furthermore, the present results and similar previous reports [17,19] clearly indicate that a part of CB cells are GABA-negative. Although, the neurotransmitters used by these neurons are unknown. However, the present results demonstrate that at least some GABA-negative cells are VGLUT2, suggesting that they may utilize glutamate. This suggestion that some CB neurons in the amygdala could be glutamatergic was originally proposed by some authors based on morphological evaluations [19,55,57]. It might be partially supported by the fact that in the basal forebrain, while most PV neurons are GABAergic, a larger portion of CB or CR cells are glutamatergic but not GABAergic [37].

The present results report that in the amygdala of the guinea pig, only half of CR neurons (~53%) co-expressed VGAT, while the percentage of these cells co-expressing VGLUT2 was usually two times larger than that for PV (~3.5%). This is quite different from the studies of Kemppainen and Pitkänen [19], who reported that in the rat basolateral amygdala, only 24% of CR neurons were GABA-positive. This study of McDonald and Mascagni [17] reported that 75% of these cells in the rat basolateral amygdala were GABA-positive. Despite this discrepancy, the present results and previous studies [17,19] indicate that many CR cells are indeed GABAergic. These cells form a separate subpopulation of the amygdala GABAergic neurons as they neither co-express PV or CB nor somatostatin and/or neuropeptide Y. However, many of them co-express vasoactive intestinal peptide and/or cholecystokinin not expressed by PV or CB cells [67,68]. Although there are no detailed synaptic studies in the amygdala focusing on the CR neurons, there is evidence that in the neocortex, such neurons are mostly dendrite-targeting interneurons, which create synapses typically on dendritic shafts and less often dendritic spines or somata [69]. Interestingly, in some cortical areas and layers, CR neurons often innervate GABAergic interneurons [70] and, in this way, exert a significant disinhibitory effect on the pyramidal neurons [69,71]. A similar disinhibitory mechanism mediated via CR neurons was also described in the hippocampus [65]. However, there are some lines of evidence that not all CR neurons in the amygdala are GABAergic interneurons. The present results and some others [17,19] indicate that a large part of CR cells is GABA-negative. This reflects the situation in the cerebral cortex [33] and hippocampus [72], where many CR cells are GABA-negative. Furthermore, there is evidence that at least some amygdala CR neurons are projecting neurons [30]. Similar cells, which contain CR and project to extrinsic areas, were also identified in the cerebral cortex [73] and hippocampus [74]. Finally, the present results provide additional evidence indicating that at least a part of CR neurons contain VGLUT2 and may use glutamate. Interestingly, neurons containing CR and VGLUT2 have been demonstrated in the cerebral cortex [33]. 

There are several issues worth discussing. In general, the mammalian amygdala is composed of quite distinct two regions, “cortex-like” and “striatum-pallidum-like” amygdala, with different embryological origins [8,9,10,11] and cellular structures [3,12]. Thus, the probable origin, chemical phenotype, and co-expression pattern of PV, CB, and CR neurons with VGAT and VGLUT2 in both these regions should be different. Indeed, the functions of both populations are different as in the “cortex-like” amygdala, the majority of PV, CB, and CR neurons are GABAergic interneurons, while in the “striatum-pallidum-like” amygdala, these GABAergic cells are usually projection neurons [8,23,24]. However, the co-expression patterns of PV, CB, and CR cells with VGAT and VGLUT2 in both these regions are very similar according to the present results. The similar pattern of co-expression of these cells may be partially explained by the fact that most GABAergic neurons of the telencephalon, including “cortex-like” and “striatum-pallidum-like” amygdala, derive from the same subpallial sources such as medial and caudal ganglionic eminences which contribute this type of cell to all amygdala nuclei [75]. However, the morphology of GABAergic neurons in both regions is extremely different [12,76], and the co-expression pattern and electrophysiological properties of CB and CR neurons in the “striatum-pallidum-like” amygdala are still obscure. Another issue is that the populations of PV, CB, and CR neurons in the amygdala, which are GABA-negative, are quite large [17,19], especially among CR cells; thus, the populations of VGLUT2 detected in the present study may form only a part of these cells. One possible explanation may be that these cells may use neurotransmitters other than GABA or glutamate. Another one may be that glutamatergic populations in the amygdala are more numerous. In the present study, only VGLUT2 was investigated, while VGLUT1 and VGLUT3 neurons are also present in the amygdala [77,78]. Interestingly, neurons containing CB and VGLUT3 were identified in the cerebral cortex and hippocampus [79]. The next issue is that in the brain, there are neurons that co-express both VGAT and VGLUT2 [50,80] and may co-release both GABA and glutamate at the same synapses [81]. The striatal efferent may contain both glutamate and GABA [82], and the hippocampal axon terminals contain both VGLUT2 synaptic vesicles and vesicles that carry VGAT [83]. Moreover, in the rat cerebral cortex and hippocampus, there are also basket cells co-expressing cholecystokinin, GAD, and VGLUT3 [79,84]. However, it is commonly believed that neurons are exclusively either GABAergic or glutamatergic [85,86]. The results presented in the amygdala of the guinea pig show that the vast majority of PV, CB, and CR neurons predominantly involve VGAT. Although there might also be a population of PV, CB, and CR neurons that use VGLUT2, these cells may use glutamate. Further research in this area should be conducted in the future.

## 4. Materials and Methods

### 4.1. Subjects

Five male adult Dunkin-Hartley guinea pigs (*Cavia porcellus*, L.) aged 3 months and weighing 0.8 kg were used in the present study. All those animals were purchased from the Nofer Institute of Occupational Medicine in Łódź (Poland) to ensure their proper parameters, i.e., the same strain, the same age, and similar weight, etc. Animal care and handling were in strict accordance with the European Union Directive for animal experiments (2010/63/EU), and all experiments were also approved by the Local Ethical Commission of the University of Warmia and Mazury in Olsztyn (No. 58/2014). In addition, all efforts were made to minimize animal suffering and to use the minimum number of animals necessary to produce reliable scientific data.

### 4.2. Tissue Preparation

Following the habituation phase, all animals were deeply anesthetized with an intraperitoneal injection of pentobarbital (Morbital, Biowet, Poland; 2 mL/kg body weight), and after cessation of breathing, immediately perfused transcardially with saline (0.9%) followed by 4% paraformaldehyde (pH 7.4; 1040051000, Sigma Aldrich, Taufkirchen, Germany) in phosphate-buffered saline (PBS; P5493, Sigma Aldrich, Taufkirchen, Germany). After perfusion, brains were carefully dissected from the skulls and post-fixed by immersion in the same fixative for 24 h, washed twice in 0.1 M phosphate buffer (pH = 7.4, 4 °C), and then stored for 3–5 days in graded solutions (19% and 30%) of sucrose (S0389, Sigma Aldrich, Saint Louis, MO, USA) in 1×PBS at 4 °C until full saturation. Finally, the brains were frozen and then coronally sectioned at a thickness of 10 μm using a cryostat (HM525 Zeiss, Germany). The sections were mounted on object slides and stored at −80 °C until further processing.

### 4.3. Immunofluorescence

Amygdala sections were processed for routine double-immunofluorescence labelling using primary antisera raised in different species and species-specific secondary antibodies (Table 4). All staining steps were always carried out in humid dark chambers (Immuno Slide Staining Trays, R64001-E, Pyramid Innovation Ltd., Polegate, UK) and at room temperature. As each slide always contained two sections, four neighboring slides (eight neighboring sections) were always used for staining with the use of 6 different primary antibodies. Six of these eight sections were used for the main experiment (co-localization of three calcium-binding proteins and two transporters), and two spare sections were used for pan-neuronal markers to visualize amygdala nuclei. Briefly, the samples were washed three times in PBS and then incubated for one hour with blocking buffer (0.1 M PBS, 10% normal donkey serum (17-000-121, Jackson ImmunoResearch, Cambridge, UK), 0.1% bovine serum albumin (A7030, Sigma Aldrich, Taufkirchen, Germany), 1% Tween (11332465001, Sigma Aldrich, Taufkirchen, Germany), 0.05% thimerosal (T5125, Sigma Aldrich, Taufkirchen, Germany), 0.01% NaN_3_ (26628-22-8, Sigma Aldrich, Taufkirchen, Germany)). The sections were then rinsed in PBS and incubated overnight with a mixture of primary antibodies, namely a combination of the appropriate primary antibody to one of the calcium-binding proteins (PV, CB, or CR) and one of the transporters (VGAT or VGLUT2). Additional incubations included neuron-specific nuclear protein (NeuN, pan-neuronal marker). The antibodies were diluted in a blocking buffer. After incubation with the primary antibodies, the sections were rinsed in PBS and incubated for one hour with a mixture of species-specific secondary antibodies. Finally, all samples were rinsed in PBS and then mounted with carbonate-buffered glycerol (pH 8.6, G9012, Sigma Aldrich, Taufkirchen, Germany), and coverslipped.

### 4.4. Controls

The specificity of the primary antisera used in this study has been shown by manufacturers and various researchers using these products in multiple previous studies [6,87,88,89,90,91,92,93,94,95]. The antibody against NeuN is an excellent marker for neurons in the central and peripheral nervous systems [87]. The mouse antibodies directed to calcium-binding proteins (anti-calbindin; 300, anti-parvalbumin; P3088, and anti-calretinin; 6B_3_) were positively evaluated by Western blotting of the guinea pig brain homogenates and immunohistochemistry in brain sections from knock-out mice proving their specificity to their targets [96,97]. The rabbit antibodies against VGAT (AB5062P) and VGLUT2 (135 402) were frequently applied in different studies [33,50,98,99,100,101,102], and they were also positively verified by Western blotting of the mice brain extracts and immunohistochemistry in brain sections from knock-out mice [103,104]. The specificity of secondary antibodies was controlled by the omission and/or replacement of all primary antisera by non-immune sera or PBS. The lack of any immunoreactions indicated specificity. 

### 4.5. Cell Counts

The co-expression pattern of PV, CB, and CR cells with VGAT and VGLUT2 were analyzed in the “cortex-like” amygdala (lateral, basolateral, basomedial, and cortical nuclei) and “striatum-pallidum-like” amygdala (central and medial nuclei) using an Olympus BX51 microscope (Olympus GmbH, Germany) equipped with Cell-F image analysis software (analySIS 5.0, Olympus Soft Imaging Solutions, Hamburg, Germany). Only the numbers of single and double-labeled PV, CB, and CR cells were counted. Single-labeled VGAT and VGLUT2 cells were excluded from the investigation. For each nucleus in each of the subjects, particular combinations of antigens were counted on seven evenly spaced sections arranged from the rostral to the caudal extent of the amygdala. To confirm the localization of the individual amygdala nuclei on the sections, neighboring sections stained with mouse anti-NeuN antibody (pan-neuronal marker) were used. All counts on the single section were performed at 40× magnification using 347.6 μm × 260.7 μm regions (test frames). The test frames were always arranged to ensure coverage of the whole cross-section area of each nucleus studied. The numbers of test frames studied per nucleus were as follows: LA: 6–8, BL: 4–6, BM: 3–5, CE: 2–4, ME: 4–6, and CO: 2–3. Within test frames, single-labeled and double-labeled neurons were counted separately. Such separate counts made within the test frames in the single nucleus in the subject were averaged. Finally, counts for each nucleus from five subjects were averaged and expressed as means ± standard error of mean (SEM). 

### 4.6. Volume Density Counting

The volume density of VGAT and VGLUT2 in the “cortex-like” and “striatum-pallidum-like” amygdala were evaluated using stained sections, according to the automated line scan analysis described and validated by Sathyanesan et al. [105]. The identical parameters were employed for the capture of the image, encompassing the duration of exposure and camera gain, thereby delivering a satisfactory grayscale dynamic range for all images gathered from diverse sections and animals. The images were saved in an 8-bit TIFF grayscale format. Mathematically, fluorescently labeled structures can be represented as curvilinear structures with local intensity variations [105]. These local intensity extremes can be detected using a Hessian matrix [106]. This approach extracts line-like information from the input image by filtering it based on the Hessian matrix. Therefore, all analyzed fluorescence images were processed using the Hessian-based filter included in the plugin called FeatureJ [107] for the NIH ImageJ software (version 1.53). According to the validation conducted by Sathyanesan et al. [105], the following parameter options were selected: the option to select the largest eigenvalue of the Hessian tensor and the option to select the absolute eigenvalue comparison. The smoothing scale factor was set to 0.5. Five lines oriented horizontally and the same number of lines oriented vertically were used to evaluate these images. With ImageJ’s “line tool,” lines were drawn through the region of interest (ROI). The amygdala nuclei were characterized by either a straight or segmented line based on their morphology. In the subsequent step, the line scans were baseline-adjusted and subsequently processed with a peak detection algorithm. The estimation of the baseline is based on the method of distinguishing peaks (signals) from the background. To determine the amount of background noise present in the line scans, the scans were consistently obtained from various locations within the section, which were characterized by the absence of an immune signal. The ultimate background value for a single section (threshold) for a peak detection algorithm was calculated based on these measurements.

### 4.7. Photomicrographic Production

High-magnification photomicrographs of immunoreactive elements were taken with a CC-12 digital camera (Soft Imaging System GMBH, Münster, Germany) on an Olympus BX51 microscope (Olympus, Hamburg, Germany). These digital images were slightly modified to optimize the image resolution, brightness, and contrast using CS4, version 11.0, software (Adobe Systems Inc., San Jose, CA, USA).

### 4.8. Statistic

In the present study, the parametric method was applied for the analysis. The calculations were performed by utilizing Statistica 13.3 software from TIBCO Software Inc. (Palo Alto, CA, USA). Statistically, significant differences between “cortex-like” and “striatum-pallidum-like” amygdala nuclei were found using the appropriate test statistics. Data from immunohistochemical studies were averaged and then examined using Shapiro–Wilk tests. Afterwards, the homogeneity of variance was tested. An independent-sample t-test with multiple comparisons was used to determine the characteristic differences between nuclei. The significance level was set at *p* ≤ 0.05. All statistical graphs presented in the study were created using GraphPad Prism 6 software (GraphPad Software, La Jolla, CA, USA). The data are presented as box-and-whisker plots. The “box” represents the median, as well as the 25th and 75th quartiles, and the “whiskers” show the 5th and 95th percentile (n = 5).

## 5. Conclusions

The results of the present study provide evidence that in the amygdala of the guinea pig, the vast majority of PV, CB, and CR neurons primarily co-express VGAT. Thus, these results confirm previous observations indicating that populations of these cells in the mammalian amygdala are primarily GABAergic. However, these results also provide the first direct evidence that at least a fraction of neurons in each of these populations is endowed with VGLUT2, suggesting that these cells may use glutamate. It is worth mentioning that the populations of PV, CB, and CR neurons that may use glutamate are most probably more numerous as in the present study, only VGLUT2 was used as a marker, while these cells may also utilize VGLUT1 and/or VGLUT3 which are also present in the amygdala.

## Figures and Tables

**Table 4:** Specification of reagents.

Antigen	Code	Clonality	Host Species	Dilution	Supplier	Location
Primary antibodies						
NEUN	MAB377	monoclonal	Mouse	1:1000	Millipore	Temecula, CA/USA
CB	300	monoclonal	Mouse	1:4000	SWANT	Bellinzona/Switzerland
PV	P3088	monoclonal	Mouse	1:6000	Sigma Aldrich	St. Louis, MO/USA
CR	6B_3_	monoclonal	Mouse	1:2000	SWANT	Bellinzona/Switzerland
VGAT	AB5062P	polyclonal	Rabbit	1:6000	Millipore	Temecula, CA/USA
VGLUT2	135 402	polyclonal	Rabbit	1:2000	Synaptic Systems	Goettingen/Germany
Secondary antibodies						
ALEXA Fluor 488 nm	A-21202	polyclonal	Anti-Mouse	1:1000	ThermoFisher	Rockford, IL/USA
ALEXA Fluor 555 nm	A-31572	polyclonal	Anti-Rabbit	1:1000	ThermoFisher	Rockford, IL/USA

## Data Availability

The data that support the findings of this study are available from the corresponding author upon reasonable request.
